# A glutamine metabolism gene signature with prognostic and predictive value for colorectal cancer survival and immunotherapy response

**DOI:** 10.3389/fmolb.2025.1599141

**Published:** 2025-05-15

**Authors:** Yinmeng Zhang, He Zhu, Jiawei Fan, Jiahui Zhao, Yan Xia, Nan Zhang, Hong Xu

**Affiliations:** Department of Gastroenterology, The First Hospital of Jilin University, Changchun, China

**Keywords:** colorectal cancer, glutamine metabolism, microbiome, tumor microenvironment, prognosis

## Abstract

**Background:**

Colorectal cancer (CRC) remains a major cause of cancer mortality, and dysregulated glutamine metabolism has emerged as a potential therapeutic target. However, the precise role of glutamine in CRC progression and treatment response remains debated.

**Methods:**

The authors collected transcriptome and microbiome information, from multiple sources to construct the GLMscore, a prognostic signature in CRC. To comprehensively characterize the biological features of GLMscore groups, the integration of transcriptomic profiling, KEGG pathway enrichment analysis, immune infiltration analysis, tumor immune microenvironment characterization, microbiome analysis, and tissue imaging were applied. Furthermore, CRC patients were stratified into GLMscore high and GLMscore low groups. The robustness of GLMscore was validated in both training and validation cohorts, and the predictive value for immunotherapy response was assessed. Finally, single-cell RNA sequencing (scRNA-seq) analysis was conducted to delineate the differences between GLMscore high and GLMscore low groups.

**Results:**

High GLMscore was associated with elevated expression of pathways related to tumorigenesis, epithelial-mesenchymal transition (EMT), and angiogenesis. Furthermore, high GLMscore patients exhibited an immunosuppressive TME characterized by increased infiltration of M0 and M2 macrophages, reduced overall immune infiltration (supported by ESTIMATE and TIDE scores), and increased expression of immune exclusion and suppression pathways. Analysis of pathological whole-slide images (WSIs) revealed a lack of intratumoral tertiary lymphoid structures (TLSs) in high GLMscore patients. The GLMscore also predicted resistance to common chemotherapeutic agents (using GDSC data) and, importantly, predicted poor response to immunotherapy in the IMvigor210 cohort. Analysis of 16S rRNA gene sequencing data revealed an enrichment of potentially oncogenic microbiota, including Hungatella and Selenomonas, in high GLMscore group. Single-cell analysis further confirmed the immunosuppressive TME and identified increased cell-cell communication between inflammatory macrophages and tumor cells in high GLMscore group.

**Conclusion:**

The authors innovatively constructed GLMscore, a robust scoring system in quantifying CRC patients, exploring the distinct biological features, tumor immune microenvironment and microbiome ecology, exhibiting high validity in predicting survival prognosis and clinical treatment efficacy.

## 1 Introduction

Colorectal cancer (CRC) is one of the most common gastrointestinal malignancies and ranks as the second leading cause of cancer-related mortality worldwide (Siegel et al., 2024). Over the past few decades, advancements in surgical techniques and adjuvant chemotherapy have significantly improved the 5-year overall survival (OS) rates for CRC patients. Nevertheless, despite appropriate surgical resection and adjuvant therapy, nearly 30% of patients experience recurrence (Cheng et al., 2022). Current treatment strategies for CRC often involve a combination of modalities, including immune checkpoint inhibitors (ICIs) combined with chemotherapy (André et al., 2020), anti-angiogenic therapy in conjunction with ICIs (Tian et al., 2023), and other experimental therapies and treatment optimization approaches. However, it is crucial to note that the vast majority of CRC patients do not benefit from targeted therapies (Han et al., 2024).

Reprogrammed energy metabolism is an emerging hallmark of cancer (Hao et al., 2016). To meet the energetic and biosynthetic demands of proliferation, growth, and metastasis, cancer cells undergo significant metabolic alterations, a key feature of which is an increased dependence on glutamine (Hao et al., 2016). Glutamine not only fuels cancer cells, but its metabolite, α-ketoglutarate (α-KG), supporting mitochondrial metabolism (Dang, 2010). These metabolites and biosynthetic precursors are essential for rapid tumor cell proliferation. In colorectal cancer (CRC), active glutamine metabolism has emerged as a promising therapeutic target. However, clinical studies investigating the significance of glutamine levels in CRC patients are currently limited.

Tran et al. reveal that, glutamine restriction in the colorectal cancer (CRC) microenvironment enhances Wnt signaling and drives tumorigenesis by decreasing intracellular α-ketoglutarate (α-KG) levels (Tran et al., 2020). Several studies have shown that decreased serum glutamine levels are associated with increased systemic inflammation and poor prognosis in CRC patients, suggesting serum glutamine as an independent prognostic biomarker for CRC progression (Li et al., 2019). Mechanistically, glutamine deprivation promotes CRC cell migration and invasion by inducing the epithelial-mesenchymal transition (EMT) process (Sun et al., 2022), while enhanced glycolysis and glutamine metabolism are linked to CRC cell proliferation, metastasis, and resistance to 5-fluorouracil (5-FU) (Duan et al., 2024). Nevertheless, a study based on the UK Biobank cohort reported an inverse association between glutamine levels and colorectal cancer risk (Rothwell et al., 2023).

In this study, we integrated transcriptomic, microbiome, and single-cell sequencing data from over 2400 CRC patients across multiple centers to construct a glutamine metabolism-based scoring model, the GLMscore. We characterized the distinct biological features and microbiome composition of patients with high and low GLMscores. Furthermore, by integrating pathological whole-slide images (WSIs), we investigated the unique immune infiltration patterns within the tumor microenvironment. These analyses were further extended to the single-cell level. The resulting GLMscore serves as an accurate prognostic biomarker for survival and a predictive model for therapeutic response, enabling precise patient stratification and providing guidance for personalized clinical treatment strategies.

## 2 Methods

### 2.1 Data collection and preprocessing

We obtained a curated set of 76 genes associated with glutamine metabolism from the Molecular Signatures Database, GO Biological Process gene set “GOBP_GLUTAMINE_FAMILY_AMINO_ACID_METABOLIC_PROCESS” (MSigDB, https://www.gsea-msigdb.org/gsea/msigdb). Transcriptomic data and corresponding clinicopathological information were extracted from multiple colorectal cancer (CRC) cohorts. The Cancer Genome Atlas CRC cohort (TCGA-CRC) served as the training dataset. Gene expression profiles and clinical follow-up data from seven independent Gene Expression Omnibus (GEO) CRC cohorts (GSE39582, GSE28722, GSE143985, GSE75316, GSE39084, GSE38832, and GSE41258) were used for external validation. Additionally, single-cell RNA sequencing (scRNA-seq) data and clinical follow-up information from 20 CRC patients were incorporated to predict immunotherapy response and characterize the immune microenvironment.

RNA sequencing data for the TCGA-CRC cohort were downloaded from the UCSC Xena database (Goldman et al., 2020), and clinicopathological data were obtained from the Supplementary Material of Liu et al. (2018). Transcriptomic data (transcripts per million, TPM), intratumoral microbiome data, and clinical follow-up data for the AC-ICAM cohort were acquired from Roelands et al. (2023). Data from the seven GEO cohorts were downloaded from the GEO database (https://www.ncbi.nlm.nih.gov/geo/) and preprocessed using the “GEOquery” R package. Single-cell sequencing data and accompanying clinical data for the 20 CRC patients were obtained from Zhang et al. (Chen et al., 2024). Immunohistochemistry (IHC) images were acquired from The Human Protein Atlas (https://www.proteinatlas.org).

### 2.2 Construction of GLMscore

To identify glutamine metabolism-related genes (GLMGs) significantly associated with prognosis, we performed univariate Cox proportional hazards regression analysis on each gene using disease-free survival (DFS) as the outcome variable, combined with a 1000-times resampling procedure (each time randomly selecting 80% of the samples). For each iteration, p-values from Cox regression were recorded for all candidate genes. We then calculated the frequency with which each gene achieved statistical significance (p < 0.05) across the 1,000 iterations. Genes that were significant in over 800 out of 1,000 iterations were considered robustly associated with DFS and were retained for further analysis. This strategy increases robustness over single-run analysis by reducing sample-dependent variability. This analysis was conducted on a training dataset. To mitigate the risk of overfitting inherent in high-dimensional data, we employed the Least Absolute Shrinkage and Selection Operator (LASSO) regression to select a subset of genes with the strongest prognostic value (“glmnet” R package, version 4.1–8). The resulting gene signature was used to construct a glutamine metabolism-based risk score (GLMscore), calculated as follows: GLMscore = ∑*i* = 1 Coefficient (GLMGi) * Expression (GLMGi). Patients were stratified into low- and high-GLMscore groups based on an optimal cutoff value determined by the Youden index.

### 2.3 Assessment of biological characteristics, immune microenvironment and microbiome ecology

To characterize the tumor microenvironment (TME) and immune infiltration, we calculated a series of gene signature scores using single-sample Gene Set Enrichment Analysis (ssGSEA) implemented in the GSVA R package. These scores were derived from the HALLMARK gene sets (h.all.v2023.1.Hs.entrez) obtained from the MSigDB (https://www.gsea-msigdb.org/gsea/msigdb/human/genesets.jsp?collection=H). Additionally, we utilized gene sets representing various TME-infiltrating immune cell types, as defined by Charoentong et al. (2017). The ESTIMATE algorithm was employed to provide an independent validation of the ssGSEA results (Yoshihara et al., 2013).

To further investigate tumor-immune interactions and assess potential immunotherapy responsiveness, we analyzed key molecules and pathways using the “IOBR” R package (Zeng et al., 2021a). This analysis included immune checkpoint genes, immune suppression signatures, immune exclusion signatures, and immune exhaustion signatures. We also evaluated established predictors of immunotherapy response, including the Tumor Immune Dysfunction and Exclusion (TIDE) score (Jiang et al., 2018) and the TMEscore (Zeng et al., 2021b). Immune cell infiltration levels were quantified using the CIBERSORT deconvolution algorithm (Newman et al., 2015). Finally, differences in the intratumoral microbiome niche between molecular subtypes were assessed using 16S rRNA gene sequencing data from the AC-ICAM cohort (Roelands et al., 2023).

### 2.4 Pathological assessment and IHC visualization

Tertiary lymphoid structures (TLSs) are ectopic lymphoid aggregates that form in non-lymphoid tissues, often in response to chronic inflammation or cancer. They are characterized by a dense, unencapsulated cluster of B cells (CD20^+^) at the periphery, an adjacent T cell zone (CD3^+^), and surrounding dendritic cells (DCs; CD11c+) (Teillaud et al., 2024).

To investigate the prognostic significance of TLSs in colorectal cancer (CRC), 598 whole-slide images (WSIs) from TCGA-CRC samples were meticulously annotated by board-certified pathologists who were blinded to the clinical outcomes. Based on their location relative to the tumor invasive margin, TLSs were classified into two subgroups, peritumoral TLSs (peri-TLSs) and intratumoral TLSs (intra-TLSs).

### 2.5 Immunotherapy response and drug sensitivity

Drug sensitivity to common chemotherapeutic agents was predicted using the Genomics of Drug Sensitivity in Cancer (GDSC) database (Yang et al., 2013). To assess potential immunotherapy response, we analyzed immune checkpoint-related genes obtained from Qin et al. (Qin et al., 2019). Furthermore, we utilized two independent immunotherapy cohorts as supplementary datasets. The IMvigor210 cohort (Mariathasan et al., 2018), comprising samples from patients with metastatic urothelial cancer treated with an anti-PD-L1 agent, was accessed via the “IMvigor210CoreBiologies” R package.

### 2.6 Microbiome analysis and single-cell analysis

The 16s rRNA microbiome data used in this study were obtained from intratumoral microbiome samples collected from patients in the AC-ICAM cohort (Roelands et al., 2023). To investigate the relationship between microbial composition and GLMscore, we compared the relative abundance of microbial species between the GLMscore-high and GLMscore-low groups. This analysis was performed at the genus level, focusing on bacterial taxa that were differentially enriched in these groups. Single-cell RNA sequencing (scRNA-seq) data analysis was performed using the Seurat R package (version 5.2.1). Expression data for the scRNA-seq cohort were obtained from Zhang et al. (Chen et al., 2024). To ensure reproducibility, we adhered to the original analytical parameters described in Zhang et al.’s literature. Cell-cell communication and intercellular ligand-receptor interactions were inferred using the “CellChat” R package.

### 2.7 Statistical analysis

All data processing and statistical analyses were conducted using R software (version 4.1.0). Comparisons between two groups were performed using the Wilcoxon rank-sum test, whereas the Kruskal–Wallis test was used for comparisons involving more than two groups. Kaplan-Meier survival curves were generated, and survival differences were assessed using the log-rank test, implemented in the “survminer” R package. Correlation analyses were performed using Spearman’s rank correlation coefficient. Statistical significance was defined as a p-value <0.05.

## 3 Results

### 3.1 Development and validation of GLMscore

**FIGURE 1:**
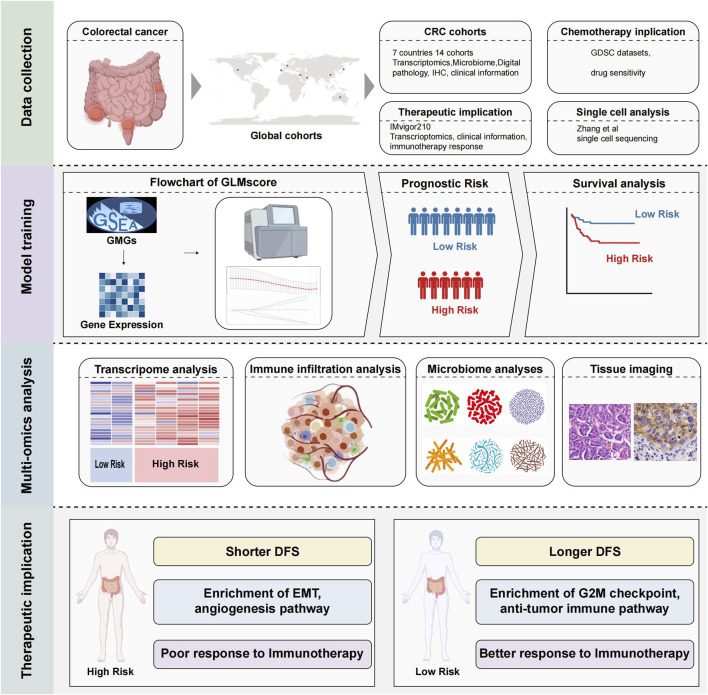
The overall workflow of this study.

**FIGURE 2:**
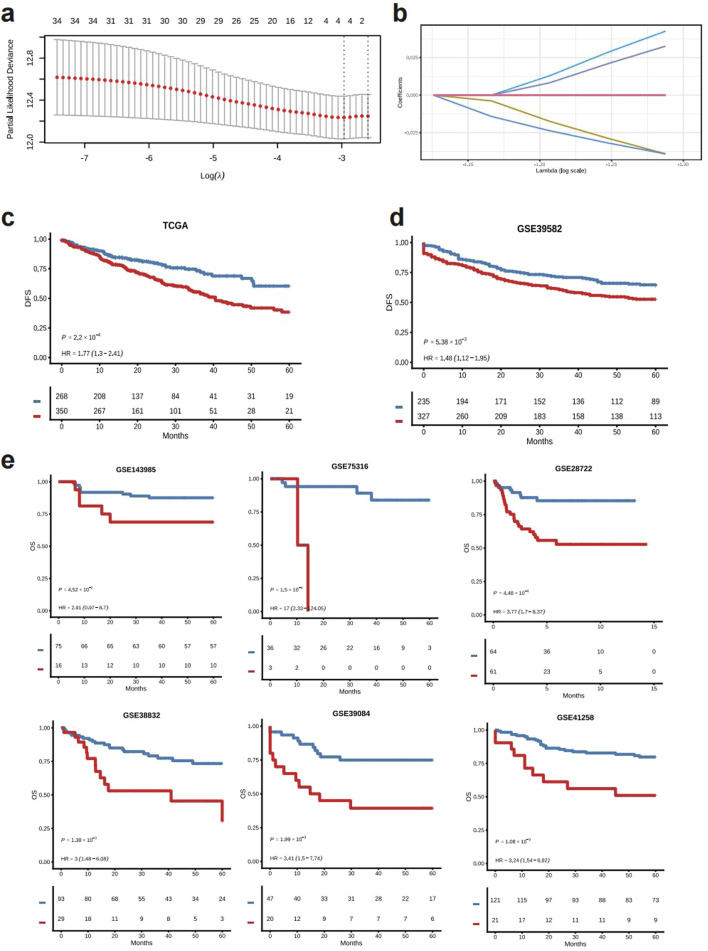
The construction and validation of GLMscore. **(a)**, The features selected by the Lasso cox model. **(b)**, The selected features and accordingly coefficients. **(c)**, Survival analysis of the training dataset (TCGA-CRC). **(d,e)**, Survival analysis of validation cohorts (GSE39582, GSE143985, GSE75316, GSE28722, GSE38832, GSE39084, GSE41258).

The comprehensive workflow is illustrated in Figure 1. A total of 76 glutamine metabolism related genes were harvested from the MsigDB, finally 35 genes were utilized in the construction of GLMscore after the resampling process. A four gene signature was developed, and the GLMscore formula is as follows: (−0.0391*ASRGL1) + (0.0426*MECP2) + (−0.0391*NOS2) + (0.0326*NOS3) (Figures 2a,b). According to the optimal cut-off value, patients were classified into two GLMscore group. Samples with higher GLMscore trended to significantly shorter DFS in both training cohort and validation cohorts (Figures 2c–e). Taking into consideration GLMscore group cases and other clinical or pathological parameters significant in univariate Cox regression, the results of multivariate Cox regression showed that GLMscore was an independent prognostic factor for DFS prediction in both training cohort and validation cohorts.

### 3.2 Biological characteristics of GLMscore groups

**FIGURE 3:**
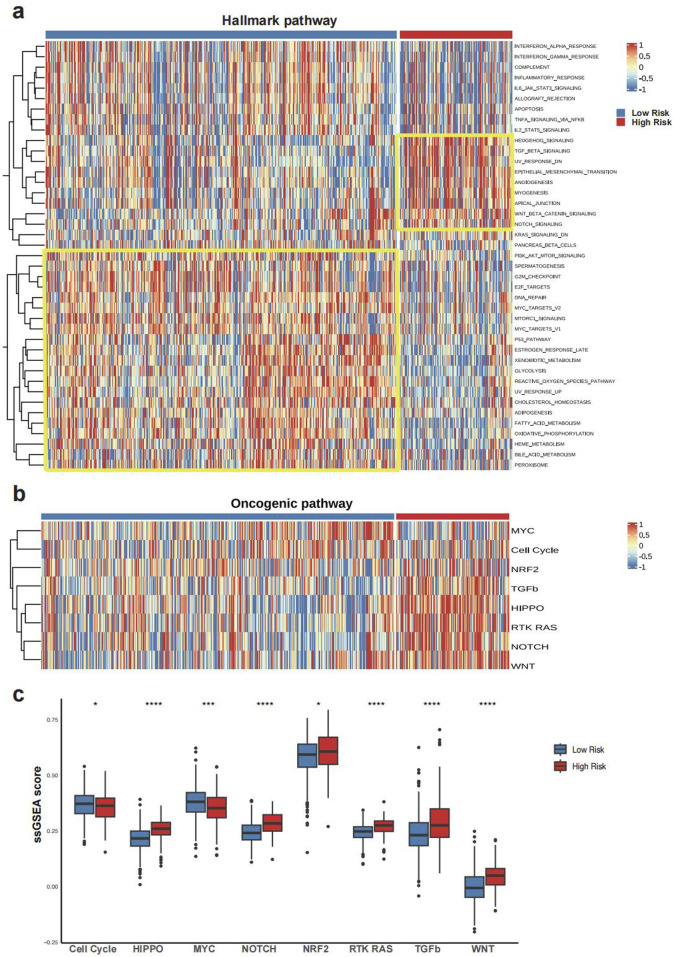
The biological features between GLMscore groups. **(a)**, Heatmap of Hallmark pathways enrichment in GLMscore groups. **(b)**, Heatmap of oncogenic pathway enrichment analysis in GLMscore groups. **(c)**, Boxplot of oncogenic pathway enrichment analysis between GLMscore groups.

Hallmark pathway enrichment analysis revealed distinct biological features between the two GLMscore groups. GLMscore low group was associated with pathways enriched in Myc-targets, E2F-targets, and G2M-checkpoint, while GLMscore high group showed enrichment in pathways related to carcinogenesis, EMT, angiogenesis, inflammation and hypoxia (Figure 3a). GLMscore high group displayed significantly elevated mutation levels in the HIPPO, NOTCH, RTK RAS, and WNT pathways, which are closely associated with cell proliferation, tumor invasion, and metastasis (Figures 3b,c).

### 3.3 Immune infiltration between GLMscore groups

**FIGURE 4:**
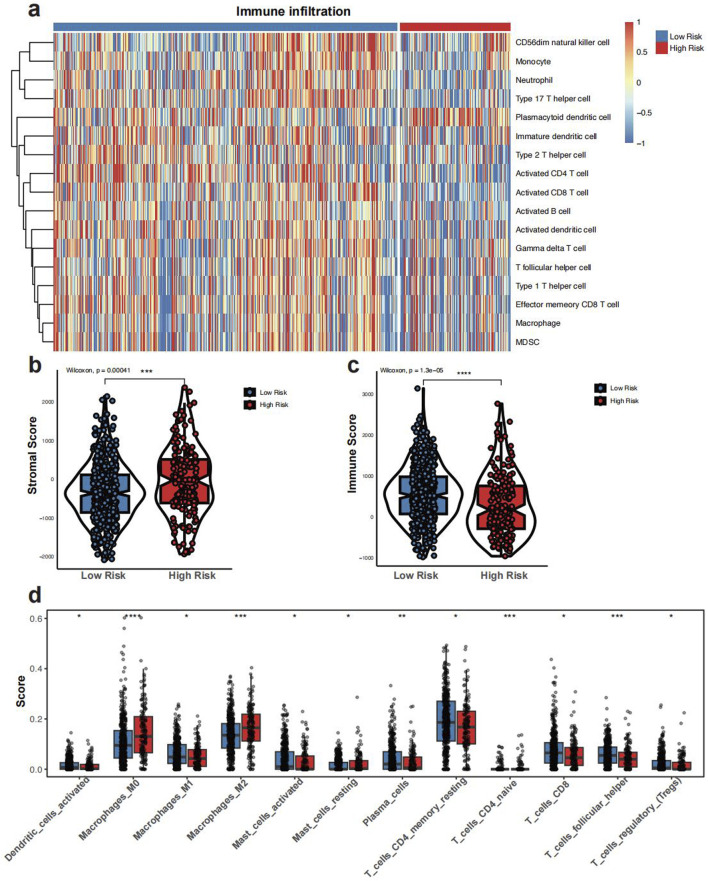
**(a)** Heatmap of immune infiltration in GLMscore groups. **(b, c)** Stromal and immune scores calculated by ESTIMATE in GLMscore groups. **(d)** CIBERSORT deconvolution analysis of immune cell infiltration between GLMscore groups.

**FIGURE 5:**
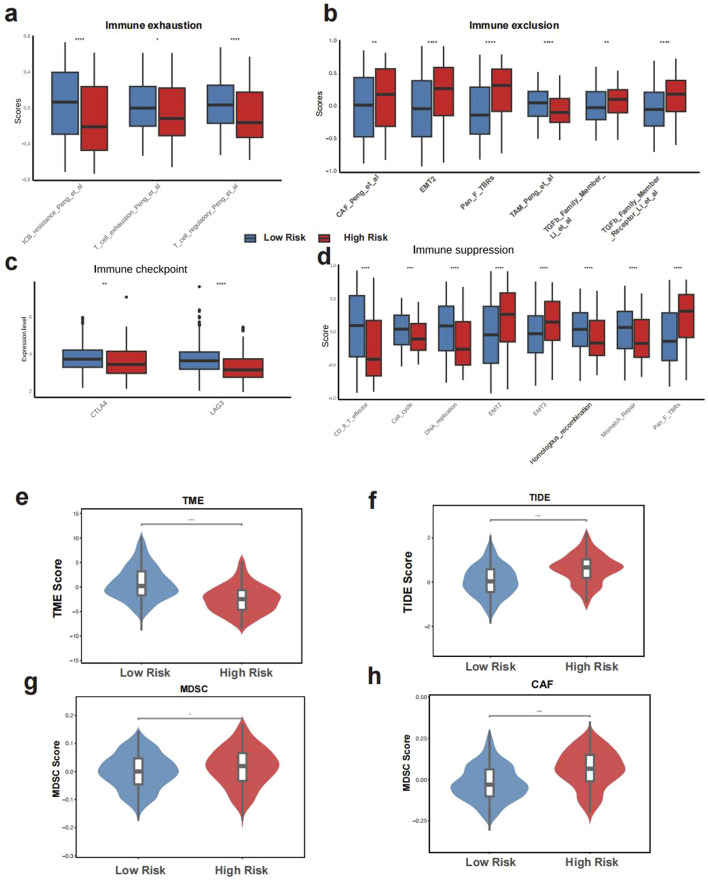
The tumor immunemicroenvironment of GLMscore groups. **(a–d)**, IOBR algorithm results of immune suppression, immune exhaustion, immune exclusion and the expression of immune checkpoint genes. **(e–h)**, TME score, TIDE score among GLMscore groups.

In the low GLMscore group, more immune cell infiltration was observed (Figure 4a). The high GLMscore group exhibited a higher stromal score, while the immune score displayed an opposite trend (Figures 4b,c). Additionally, the high GLMscore group was correlated with significantly more macrophages M0 and M2 infiltration in the tumor microenvironment using CIBERSORT deconvolution method (Figure 4d). According to the results of IOBR algorithm, the high GLMscore group showed a higher immune exclusion, suppression state, while the expression of immune checkpoint genes and immune exhaustion were higher in the low GLMscore group (Figures 5a–d). The TME score was higher in the low GLMscore group (Figure 5e). In contrast, the TIDE score was significantly higher in the high GLMscore group, accompanied with elevated MDSC and CAF score (Figures 5f–h).

### 3.4 Immunotherapy response and chemotherapy sensitivity

**FIGURE 6:**
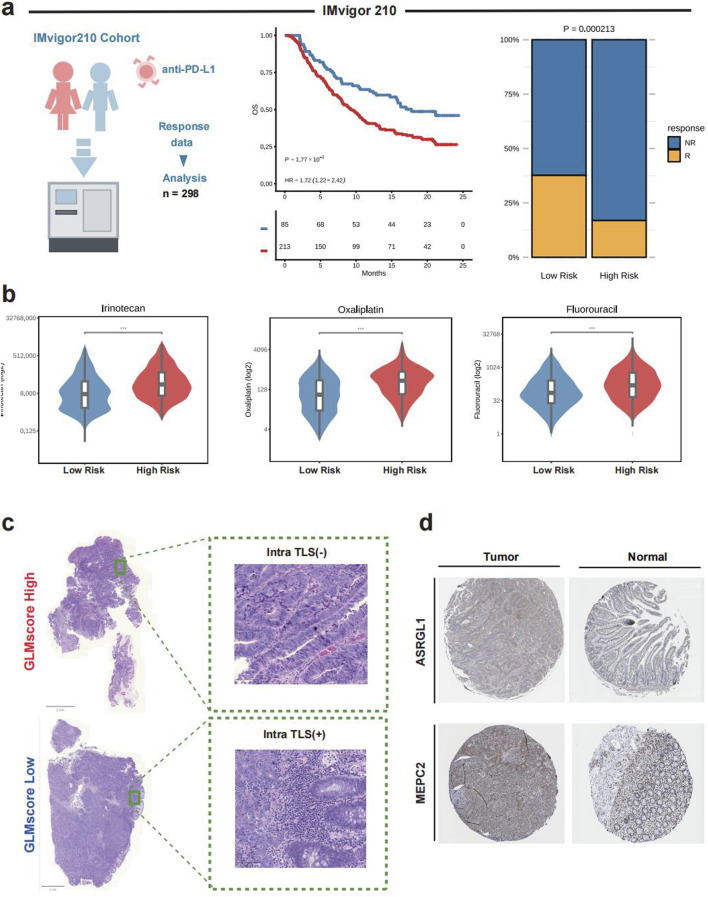
Immunotherapy response and drug sensitivity. **(a)**, Prognosis and immunotherapy response prediction in the IMvigor210 cohort. **(b)**, Drug IC50 in GLMscore groups. **(c)**, Representative WSI images of GLMscore high and low group. **(d)**, IHC images of features selected by the Lasso cox model expression of normal and tumor tissues.

The high GLMscore group exhibited a lower OS rate in the IMvigor 210 cohort, with a significantly higher proportion of non-responders (Figure 6a). Besides, the GLMscore high group displayed higher IC50 in Irinotecan, Oxaliplatin and Flurouracil, indicating worse chemotherapy sensitivity (Figure 6b). Besides, the high GLMscore group was found without intraTLS according to the WSI images, while an intraTLS was observed in the low GLMscore group (Figure 6c). Moreover, the IHC images in tumor and normal tissues revealed that ASRGL1 and MECP2 were highly expressed in tumor (Figure 6d).

### 3.5 Microbiome characteristics and single cell analysis

**FIGURE 7:**
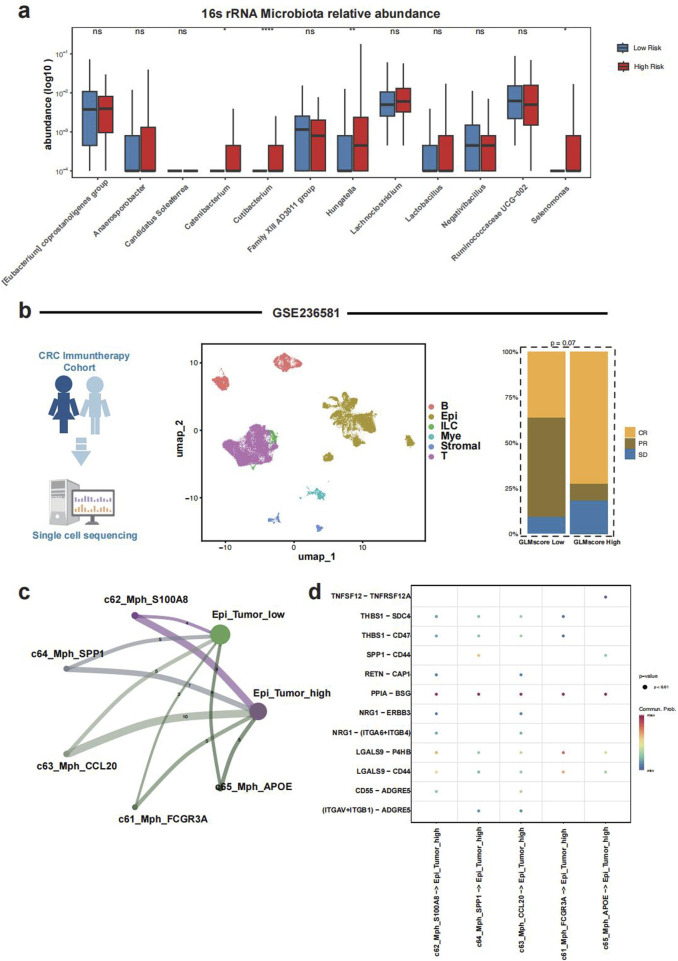
Microbiome composition and single cell analysis. **(a)**, 16s rRNA microbiome composition between the two GLMscore groups. **(b)**, Single cell analysis of Zhang et al.’s literature and the prediction of immunotherapy response. **(c)**, Cell-cell communications and ligand-receptor analysis. **(d)** Ligand -receptor interactions between macrophage subsets and GLMscore-high tumor cells.

The relative abundance of pathogenic microbiota including Hungatella and Selenomonas was significantly higher in the GLMscore group (Figure 7a). Besides, the abundance of Catenibacterium and Cutibacterium was also higher in the GLMscore group (Figure 7a). According to the single cell analysis in Zhang’s et al. research, the GLMscore high group exhibited a higher proportion of SD patients, which indicated a worse immunotherapy response (Figure 7b). The tumor cells were further subgroup into tumor high and tumor low utilizing the GLMscore formula, the SPP1+ and CCL20+ macrophages shared elevated intercellular communications with tumor high cells (Figure 7c). The ligand receptor analysis indicated that the potential signaling pathway linking the macrophages and tumor cells was the PPIA-BSG (Figure 7d).

## 4 Discussion

Colorectal cancer (CRC) is a leading cause of cancer incidence and mortality worldwide, with over 1.9 million new cases and approximately 940,000 deaths reported in 2020. Global incidence is projected to rise to 3.2 million new cases by 2040 (Xi and Xu, 2021). Despite advancements in treatment over the past decades that have improved overall survival (OS) rates in CRC, a substantial proportion of patients experience disease recurrence or exhibit resistance to chemotherapy, highlighting the persistent challenges in clinical management (Mangone et al., 2022). The role of glutamine metabolism in CRC has garnered increasing attention, as altered cellular metabolism profoundly influences cancer initiation and progression. However, the precise role of glutamine metabolism in CRC remains controversial. While some studies suggest that elevated glutamine metabolism promotes CRC development and metastasis, others, based on large-scale public databases such as the UK Biobank, have reported an inverse association between glutamine levels and CRC risk (Rothwell et al., 2023). Moreover, studies have shown that mutant KRAS alters the basal metabolism of cancer cells, increasing glutamine utilization to support proliferation. This alteration in glutamine metabolism is particularly relevant in colorectal cancer (CRC), where glutamine-dependent metabolic reprogramming plays a critical role in supporting the rapid proliferation and survival of cancer cells. KRAS mutations are common in CRC and have been linked to enhanced glutamine consumption (Najumudeen et al., 2021). Therefore, to elucidate the specific role of glutamine metabolism in CRC and to better inform clinical management, the development of a prognostic model based on glutamine metabolism is warranted. Such a model could enable precise patient stratification and, through the exploration of associated biological features, immune infiltration, and microbiome ecology, provide novel insights and guidance for clinical treatment.

In our study, we integrated transcriptomic, microbiome, and single-cell sequencing data from over 2400 CRC patients across multiple centers to construct a glutamine metabolism-based scoring model, termed the GLMscore. The GLMscore, comprising four genes, demonstrated robust and significant prognostic value for survival in both the training cohort and seven independent validation cohorts. We found that patients with high GLMscores exhibited elevated expression of pathways associated with tumorigenesis, epithelial-mesenchymal transition (EMT), and angiogenesis, partially explaining the poorer clinical outcomes observed in this subgroup. Analysis of the tumor immune microenvironment (TME) revealed that high GLMscore group possessed increased infiltration of M0 and M2 macrophages, indicative of an immunosuppressive, pro-tumorigenic TME. Further supporting this, ESTIMATE scores and TIDE scores were elevated in the high-GLMscore group, suggesting reduced immune infiltration and potential resistance to immunotherapy. Consistently, analysis using the “IOBR” R package (version 0.99.8) demonstrated significantly increased expression of immune exclusion and immune suppression pathways in the high GLMscore group. In the IMvigor210 cohort, a melanoma immunotherapy cohort, patients with high GLMscores exhibited poorer overall survival (OS) and a significantly lower proportion of responders to immunotherapy. Interestingly, studies have highlighted a connection between ASRGL1 expression and hormone receptor status, establishing a link between glutamine and amino acid metabolism and hormonal homeostasis (Zhai et al., 2023). Another crucial gene selected by the GLMscore, NOS2, plays a critical role in arginine metabolism, converting arginine into citrulline and nitric oxide (NO). While NOS2 is not directly involved in glutamine metabolism, the production of NO through this pathway can influence metabolic reprogramming in tumors, which may indirectly impact glutamine metabolism by modulating immune responses and amino acid availability (Palmieri et al., 2020).

We further investigated tertiary lymphoid structures (TLSs), ectopic lymphoid aggregates implicated in anti-tumor immunity, within the CRC microenvironment. Prior studies have shown that the presence of intra-TLSs is associated with improved survival outcomes in CRC (Lei et al., 2024). Analysis of TCGA pathological WSIs revealed a lack of intra-TLSs in the high GLMscore group, whereas classical intra-TLS structures were observed in the low GLMscore group. Consistent with these findings, analysis of drug sensitivity data from the GDSC database revealed reduced sensitivity to three common chemotherapeutic agents in the high GLMscore group.

Furthermore, analysis of 16S rRNA gene sequencing data from the AC-ICAM cohort revealed a significantly higher relative abundance of several known oncogenic bacteria, including Hungatella (Qin et al., 2024) and Selenomonas (Bullman et al., 2017), in the high GLMscore group. Elevated abundance of Catenibacterium was found to be associated with colorectal adenomas (Bosch et al., 2022), was consistent with our study results. Additionally, microbiota with previously unknown roles in CRC, such as Cutibacterium, was also significantly more abundant in the high-GLMscore group, suggesting a potential pathogenic microbiota in CRC. These findings suggest that an increased abundance of potentially oncogenic microbiota may contribute to CRC development in the high GLMscore subgroup.

Finally, our study explored the TME characteristics and immunotherapy response at the single-cell level. We observed a higher proportion of patients with a stable disease (SD) status in the high GLMscore group compared to the low GLMscore group. The GLMscore demonstrated strong predictive accuracy for immunotherapy response, with validation performed in both a single-cell CRC immunotherapy cohort and an external immunotherapy cohort. This reinforces the utility of the GLMscore as a reliable biomarker for predicting response to immunotherapy in colorectal cancer (CRC). The validation across two independent cohorts further supports its robustness and potential clinical applicability. Notably, the association between GLMscore and immune-related markers highlights the potential of glutamine metabolism as a key player in the tumor-immune interaction, which could guide personalized immunotherapy strategies. Furthermore, inflammatory macrophages, such as SPP1+ and CCL20+ macrophages, exhibited increased cell-cell communication with tumor cells in the high GLMscore group, potentially mediated by the PPIA-BSG signaling pathway.

In summary, this study integrated multi-omics data to develop the GLMscore, a novel scoring system for stratifying CRC patients and guiding clinical treatment decisions. We comprehensively characterized the biological features, tumor immune microenvironment, microbial composition, pathological characteristics, and IHC profiles associated with glutamine metabolism in CRC, as reflected by the GLMscore. While our study provides valuable insights, several limitations should be acknowledged. First, as a multi-center, large-scale retrospective study, further prospective validation is needed to confirm the role of glutamine metabolism in CRC. Second, although we integrated transcriptomic, microbiome, and single cell sequencing data to explore the underlying mechanisms, experimental validation is required to confirm these findings. Third, the microbiome analysis relies on 16S rRNA sequencing from a single cohort, which has limitations, including a lack of strain-level resolution and functional data. WGS or metagenomics would offer a more comprehensive view and help establish causality in CRC development.

In conclusion, our study, centered on the GLMscore, elucidates the multifaceted role of glutamine metabolism in CRC initiation and progression. Moreover, we demonstrate the robustness and accuracy of GLMscore as a prognostic biomarker for survival and, importantly, its ability to predict response to immunotherapy, offering valuable insights and guidance for clinical management.

## 5 Conclusion

This study developed and validated a robust GLMscore for patient stratification in CRC and comprehensively investigated the role of glutamine metabolism, including its impact on biological characteristics, the tumor immune microenvironment, and microbial composition, supported by pathological and immunohistochemical (IHC) visualization, offer novel insights for precision clinical management.

## Data Availability

The datasets presented in this study can be found in online repositories. The names of the repository/repositories and accession number(s) can be found in the article/Supplementary Material.
